# Generation of IgM^+^ B cell-deficient Atlantic salmon (*Salmo salar*) by CRISPR/Cas9-mediated IgM knockout

**DOI:** 10.1038/s41598-025-87658-5

**Published:** 2025-01-28

**Authors:** Mari Raudstein, Ma. Michelle D. Peñaranda, Erik Kjærner-Semb, Søren Grove, H. Craig Morton, Rolf Brudvik Edvardsen

**Affiliations:** Institute of Marine Research, Bergen, Norway

**Keywords:** Adaptive immunity, Gene editing, Aquaculture, Vaccines, Immunoglobulin, IgT, Animal biotechnology, Functional genomics, Biotechnology, Immunology, Adaptive immunity

## Abstract

Infectious diseases pose significant challenges to Norwegian Atlantic salmon aquaculture. Vaccines are critical for disease prevention; however, a deeper understanding of the immune system is essential to improve vaccine efficacy. Immunoglobulin M (IgM) is the main antibody involved in the systemic immune response of teleosts, including Atlantic salmon. In this study, we used CRISPR/Cas9 technology to knock out the two IgM genes in Atlantic salmon. High-throughput sequencing revealed an average mutagenesis efficiency of 97% across both loci, with a predominance of frameshift mutations (78%). Gene expression analyses demonstrated significantly reduced membrane-bound IgM mRNA levels in head kidney and spleen tissues. Flow cytometry revealed a 78% reduction in IgM^+^ B cells in peripheral blood, and Western blot analyses showed decreased IgM protein levels in serum. Notably, an upregulation of IgT mRNA was observed, suggesting a potential compensatory mechanism. This work presents the first application of CRISPR/Cas9 to disrupt an immune-related gene in the F0 generation of Atlantic salmon, and lays the foundation for generating a model completely lacking IgM^+^ B cells which can be used to study the role of B cells and antibodies. This study has implications for advancing immune research in teleosts and for developing strategies to improve salmon health and welfare in aquaculture.

## Introduction

Infectious diseases constitute a major challenge in the Norwegian Atlantic salmon aquaculture industry, causing economic losses and reduced animal welfare^1^. To mitigate disease outbreaks, vaccination has become the primary prophylactic measure in aquaculture. Although vaccines against bacterial pathogens generally offer robust protection, some bacterial infections remain uncontrolled through vaccination^1^. Additionally, viral vaccines often provide suboptimal protection^2^. Increasing vaccine efficacy and developing novel vaccines that protect against a broader range of pathogens will both contribute to further improvements in fish health. Therefore, a deeper understanding of the immunological processes elicited by exposure to pathogens or vaccines will be important for efforts to develop efficient vaccines. Teleost fish exhibit most of the key elements in the adaptive immune system that can provide long-term immunity to pathogens. However, with nearly 30,000 species in the superorder, fundamental differences exist between teleosts^3,4^ and it is important to study each species separately and in detail. Furthermore, because of the whole-genome duplication event in the last common ancestor of salmonids^5^, species-specific knowledge regarding the Atlantic salmon immune system is needed.

Gene-edited animals featuring specific gene knockouts are powerful tools to understand gene function or disease mechanisms. Multiple gene-edited animal models have been established for this purpose, e.g., mice^6,7^, rats^8,9^, and zebrafish^10,11^. The Clustered Regularly Interspaced Short Palindromic Repeats (CRISPR)/CRISPR-associated (Cas) system has emerged as an efficient technology for precise gene editing^12^. Here, a synthetic guide RNA (gRNA) sequence is combined with a Cas nuclease to induce precise alterations to genomic DNA. CRISPR/Cas gene editing has already been applied in Atlantic salmon to study genes and traits important in aquaculture, such as reproduction or fatty acid synthesis^13–15^, but there has to our knowledge not yet been any application of this technology on immune-related genes in this species.

Antibodies, or immunoglobulins (Igs), are key components of both the innate and the adaptive immune system and consist of variable and constant regions. Three classes of Igs have been identified in Atlantic salmon, IgM, IgT, and IgD^16^. IgM is the most abundant Ig, playing a central role in systemic and mucosal immune responses, while IgT is predominantly specialized for mucosal immunity. In contrast, IgD remains less studied and its functions are poorly understood. The genes encoding these Igs are located in two highly similar Ig heavy (IgH) chain regions, termed IgH A and B, on chromosomes 6 and 3, respectively. Similar to findings in zebrafish and rainbow trout, the Ig genes are organized with VDJ segments located upstream of the heavy chains for IgM and IgD, while IgT has its own exclusive set of VDJ segments^17,18^. This gene organization results in fish B cells expressing either IgM and/or IgD, or IgT on a single cell^19^.

IgM is the main systemic Ig^16^ and exists in two forms: a monomeric, transmembrane-bound form expressed on the surface of B cells as part of the B cell receptor (BCR) complex, which also includes the CD79α and CD79β signalling molecules, and a predominately tetrameric form found in serum. The constant region of secreted IgM consists of four Ig domains which are encoded by separate exons of the IgM heavy chain (µ1-µ2-µ3-µ4), while splicing of the transmembrane domain directly to the µ3-encoded domain generates the membrane-bound form of the protein^3,20^. Antigen-binding to membrane-bound IgM molecules within the BCR, together with co-stimulatory signals, leads to B cell activation. This activation is essential for the proliferation and differentiation of B cells, ultimately leading to the production of soluble antibodies specific to the pathogen. In mammals, IgM is considered essential for the development of functional B cells and in mammalian models where IgM was targeted for knockout, the animals were reported to be completely B cell-deficient^21,22^. However, salmon, and teleosts in general, are different from mammals and there is still limited knowledge regarding the B cell development and antibody responses following infection and vaccination in this species^23^.

In the present study, we applied CRISPR/Cas9 gene editing technology to knock out the two IgM genes in Atlantic salmon. High-throughput sequencing was used to assess the mutagenesis efficiency. To assess whether disruption of the DNA affected mRNA and protein levels, we used RT-qPCR to measure the relative gene expression, flow cytometry to determine the presence of surface IgM (IgM^+^ B cells) in peripheral blood leukocytes, and finally, Western blot to detect IgM protein (IgM antibodies) in serum. To our knowledge, this is the first report of an immune gene-specific knockout in Atlantic salmon. Employing CRISPR/Cas technology to specifically target the IgM genes in this species allows us to further elucidate the role of salmon B cells and antibodies in combating pathogens, and opens for opportunities to distinguish between antibody-mediated and non-antibody-mediated protection.

## Results

### Fish survival and growth

CRISPR/Cas9 was used to generate IgM crispant fish by co-injecting gRNAs targeting both IgM loci in salmon, and the pigmentation gene *solute carrier family 45 member 2* (*slc45a2*). Targeting *slc45a2* enables visual identification of gene-edited individuals as disruption of this gene results in a complete albino or albino-mosaic pigmentation phenotype^24^. Mutagenesis of *slc45a2* therefore serves as an indicator that the delivery of the injection mix has been successful, and that gene editing has taken place effectively^13^. Two control groups were also included, one injected with gRNA targeting *slc45a2* only (albino control) and one non-injected group (wild-type control). To assess potential detrimental effects of editing IgM, mortality was recorded in the IgM crispant group, as well as in the control groups. There were no significant differences in mortality between any of the groups. However, it is important to note that the mortality data for the IgM crispant group includes individuals that were not successfully gene-edited (i.e., pigmented fish), which are effectively wild-type. Since these fish represent a large proportion of the group, they could mask mortality specifically associated with successful IgM knockout.

**Fig. 1:**
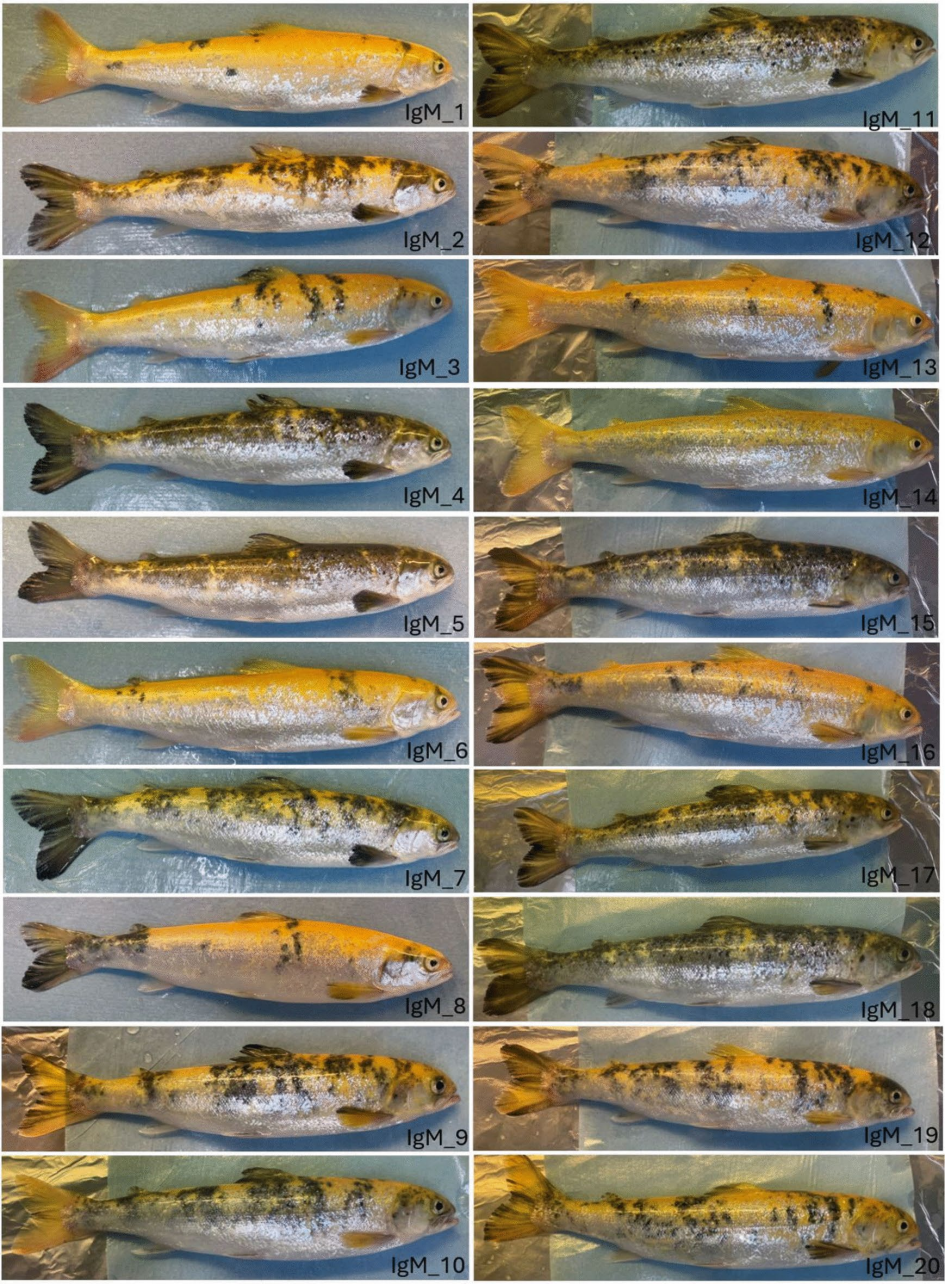
IgM crispants (IgM_1 to IgM_20) with an albino or albino-mosaic pigmentation phenotype.

For sampling, 20 fish from the IgM crispant group displaying an albino or mosaic pigmentation phenotype (Fig. 1) were selected for downstream analyses of the potential knockout effects, along with 10 albino and 10 wild-type control fish. The weight and length of each individual were recorded, and no significant differences were observed between the three groups (Table 1, Supplementary Table S1).

**Table 1:** Average weight and length of IgM crispants, albino (Alb) controls and wild-type (WT) controls.

	Length (cm)	Weight (g)
WT controls (n = 10)	23.3	144.3
Alb controls (n = 10)	25.7	188.3
IgM crispants (n = 20)	25.4	192.8

### DNA mutation analysis at IgM A and B loci

**Fig. 2:**
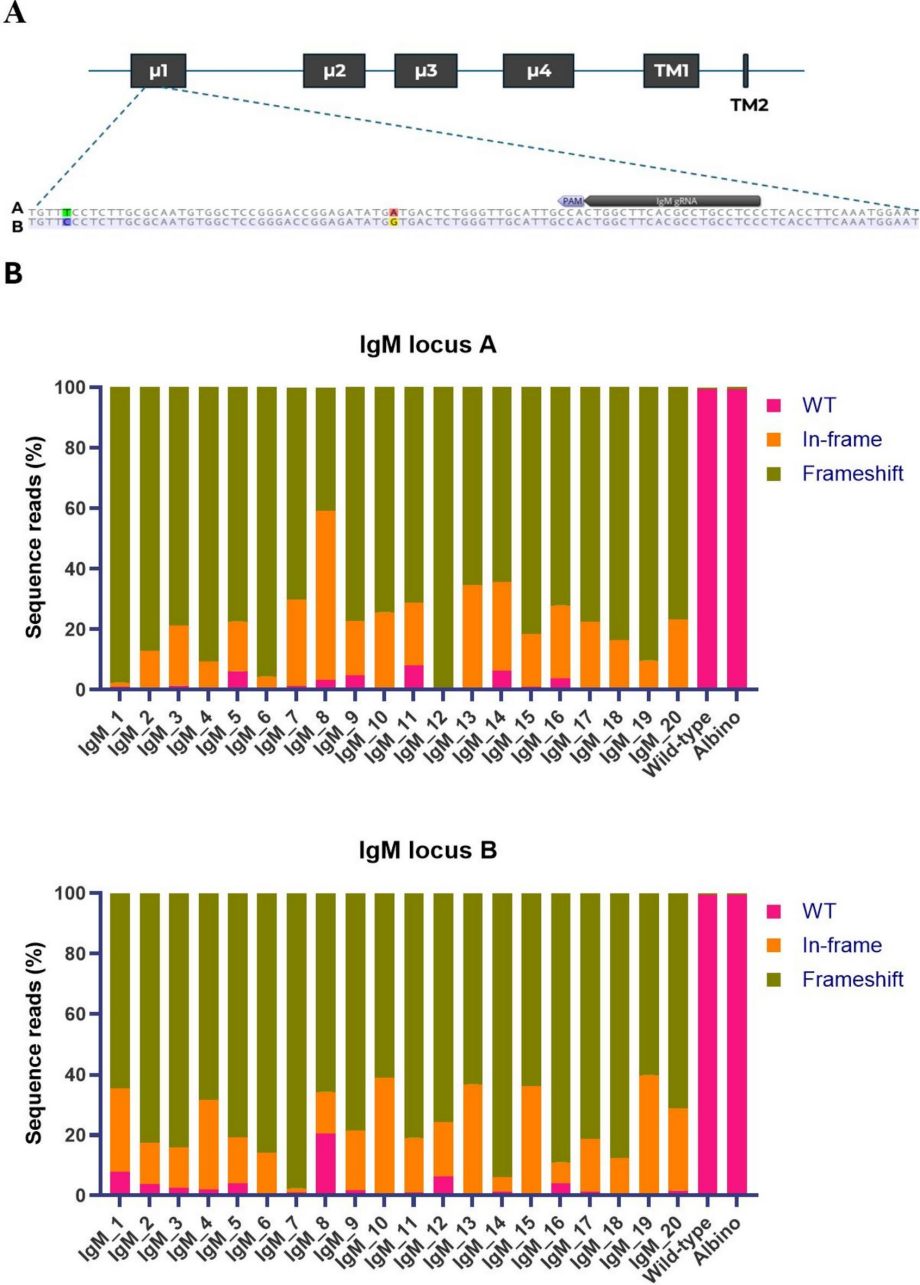
CRISPR/Cas9-mediated knockout of IgM in Atlantic salmon. (**A**) Schematic representation of the IgM locus, consisting of four exons encoding the constant domains (µ1–µ4) and two exons encoding transmembrane domains (TM1 and TM2). The gRNA target site is in the µ1 exon of both IgM A and B loci. (**B**) Mutagenesis was assessed by high-throughput sequencing of DNA obtained from fin tissue of 20 IgM crispants (IgM_1 to IgM_20). One non-edited wild-type and one albino control fish are included for comparison. The figure shows the percentage of sequence reads that contain either wild-type DNA (WT), in-frame, or frameshift mutations in IgM locus A (top) and IgM locus B (bottom).

While the pigmentation phenotype provides a visual indication of the extent of gene editing, high-throughput sequencing offers a more precise assessment of mosaicism within each individual. DNA extracted from fin tissue was sequenced to evaluate the CRISPR-induced mutagenesis at the IgM loci in 20 IgM crispants and two control fish. Screening was performed across the gRNA target site within the µ1 exon (Fig. 2A) using locus A- and locus B-specific primers. High mutation efficiencies were observed at both loci in all the fish screened (Fig. 2B, Supplementary Table S2). On average, 98% of the sequence reads obtained from locus A, and 97% of the reads from locus B, showed mutations. The impact of these mutations on the gene and resulting protein varies depending on the mutation type. Frameshift mutations, which alter the reading frame, generally disrupt the gene sequence and lead to a nonfunctional protein. In our data, the frameshift mutations predominated, accounting for an average of 79% of the sequence reads obtained from locus A, and 77% from locus B.

**Fig. 3:**
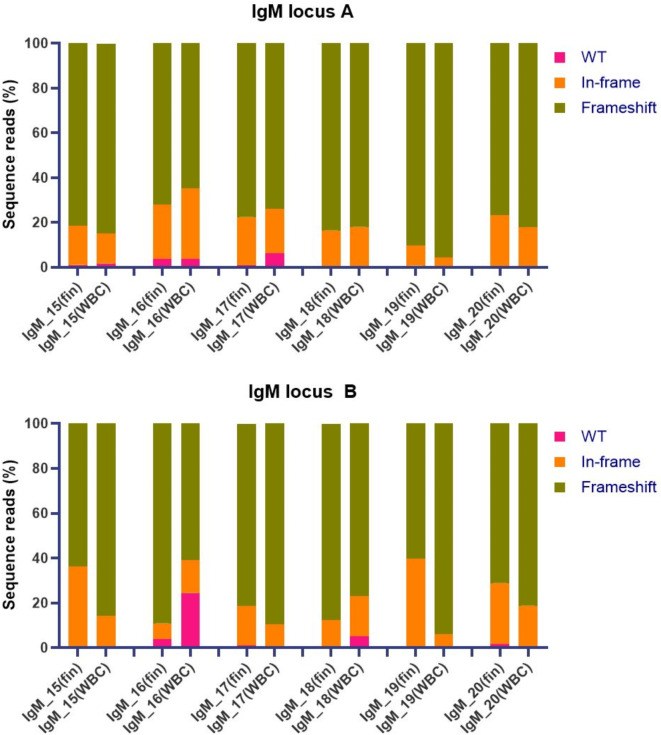
Comparison of CRISPR/Cas9-induced mutagenesis of IgM in fin and leukocyte tissues. Mutagenesis was assessed by high-throughput sequencing of DNA obtained from fin tissue (fin) and leukocytes (white blood cells, WBC) from six IgM crispants (IgM_15 to IgM_20). The figure compares the percentage of sequence reads that contain either wild-type DNA (WT), in-frame, or frameshift mutations in IgM locus A (top) and IgM locus B (bottom) in the two tissues.

We normally sample fin tissue because it is easy to collect and provides a good indication of the mutation efficiency. However, the mutation variants may differ between tissues due to mosaicism. To address this issue, we also sequenced leukocytes isolated from the blood of six IgM crispants (Fig. 3, Supplementary Table S3). The mutation efficiencies in fin tissue and leukocytes from the same individual were largely consistent for both IgM A and B loci with a few exceptions. For instance, at IgM locus B, 24% of the sequence reads obtained from the leukocytes of individual IgM_16 were wild-type, whereas only 4% of the reads from fin tissue were wild-type.

### Immunoglobulin mRNA gene expression in head kidney and spleen

**Fig. 4:**
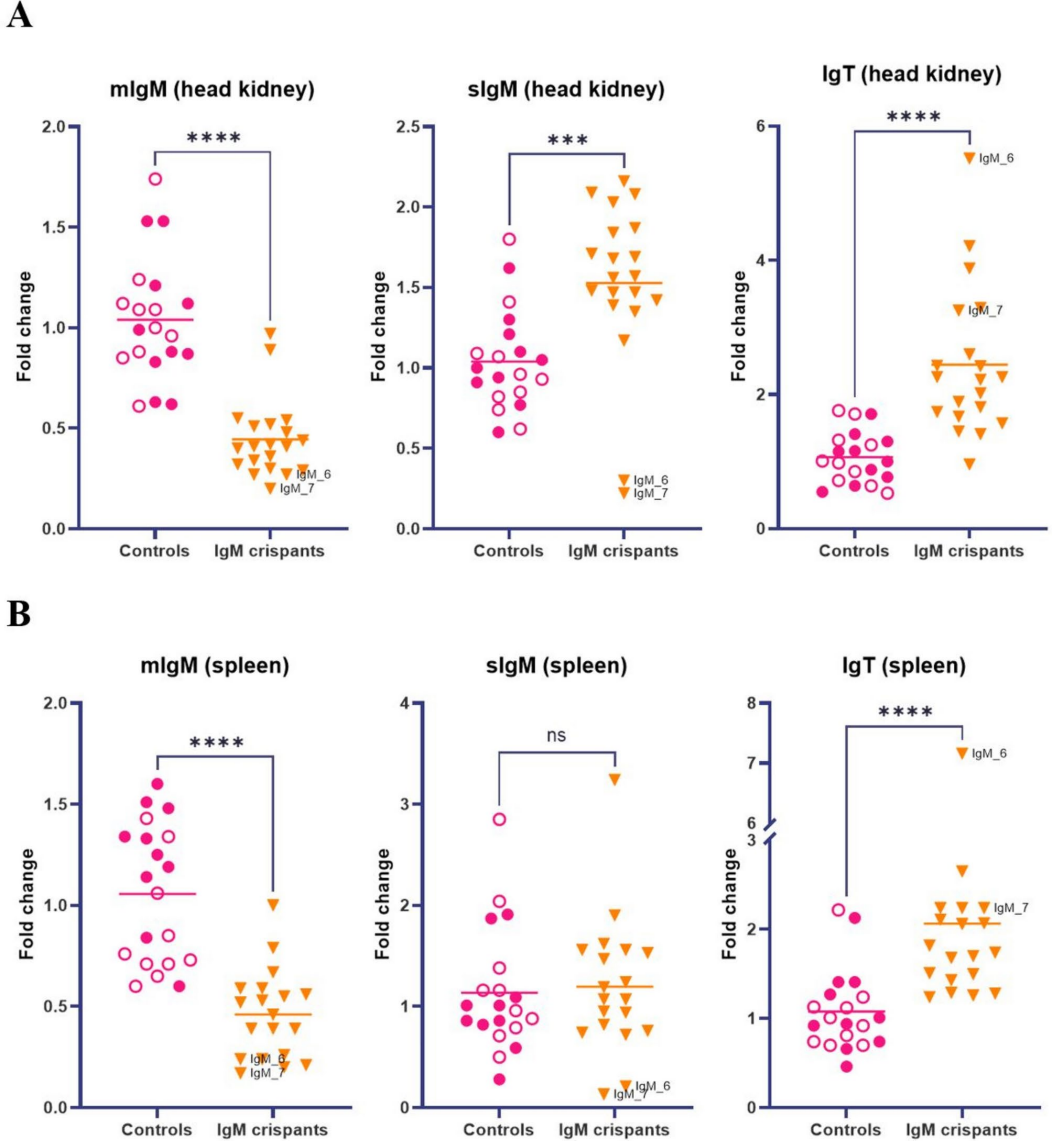
Gene expression analysis in head kidney and spleen tissues of Atlantic salmon using RT-qPCR. (**A**) Expression of membrane-bound IgM (mIgM), secreted IgM (sIgM), and IgT analysed in head kidney tissue obtained from IgM crispants (n = 20) and controls (n = 20). Wild-type controls are represented by filled circles, and albino controls by open circles. (**B**) Expression of mIgM, sIgM, and IgT analysed in spleen tissue from IgM crispants (n = 19) and controls (n = 20). Wild-type controls are represented by filled circles, and albino controls by open circles. The Mann–Whitney test was used for statistical analysis, ns = non-significant, ***p < 0.0003, ****p < 0.0001.

To investigate potential changes in the expression of Ig genes, RT-qPCR analysis was performed on head kidney and spleen tissue obtained from 19 or 20 fish from the IgM crispant group and 20 fish from the two control groups. The gene expression of membrane-bound IgM (mIgM), secreted IgM (sIgM), and IgT was measured (Fig. 4, Supplementary Table S4). It should be noted that there was a significant difference (*p* < 0.05) between wild-type and albino controls for the mIgM spleen data set (Supplementary Fig. S1). All other data sets had no significant differences between the control groups. Separating sIgM from mIgM was achieved by using qPCR primers targeting the µ3 and µ4 exons for sIgM, and µ3 and transmembrane domain exons for mIgM. The relative mIgM mRNA expression was significantly lower in both tissues of the IgM crispants compared to controls. However, while the overall sIgM mRNA expression in the spleen of IgM crispants was similar to that of control fish, and in the head kidney it was higher, two individuals (IgM_6 and IgM_7) showed notably lower sIgM mRNA levels (Fig. 4). Interestingly, IgT mRNA expression was higher in both tissues of the IgM crispants compared to controls.

### Detection of IgM^+^ B cells in blood leukocytes and total IgM in serum

**Fig. 5:**
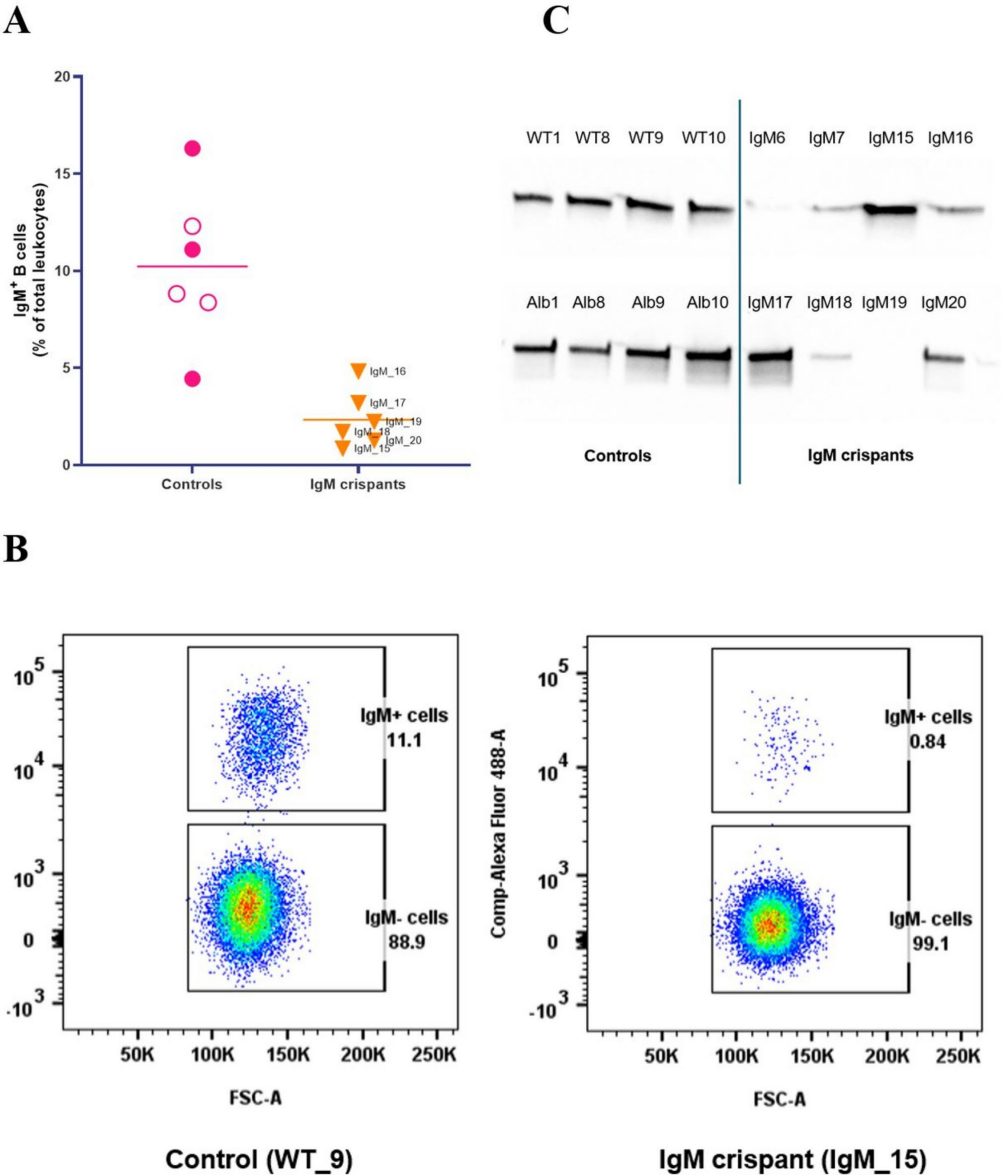
Detection of IgM protein in IgM crispants and control fish. (**A)** Peripheral blood leukocytes isolated from IgM crispants (n = 6) and controls (n = 6) were stained using an anti-IgM monoclonal antibody (F1-18), and the frequency of IgM^+^ B cells was measured using flow cytometry. Wild-type controls are represented by filled circles, and albino controls by open circles. (**B)** Representative flow cytometry data of one control (WT_9) and one IgM crispant (IgM_15). **C** IgM protein was measured in sera of controls (n = 8) and IgM crispants (n = 8) by Western blot analysis using the F1-18 antibody.

To assess whether the knockout had any phenotypic effects at the protein level, flow cytometry was performed to detect the percentage of IgM^+^ B cells in leukocytes isolated from the peripheral blood of six IgM crispants and six controls. The leukocytes were stained with an IgM-specific monoclonal antibody (F1-18), targeting the domain encoded by the µ3 exon of both IgM loci A and B^25^. A clear difference in the number of IgM^+^ B cells was observed between the IgM crispants and the control fish (Fig. 5A, B, Supplementary Table S5). In the control group, an average of 10.2% of leukocytes were IgM^+^ B cells, with individual fish ranging from 4.4 to 16.3%. In contrast, the IgM crispants showed a distinct reduction, with an average of 2.3% IgM^+^ B cells, ranging from 0.8 to 4.8% among individual fish.

The IgM protein was further examined by subjecting serum samples to Western blot analysis using the F1-18 antibody. Sera from the six fish previously analysed by flow cytometry (IgM_15 to IgM_20), along with two additional highly mutated fish (IgM_6 and IgM_7) were compared to eight controls (Fig. 5C, Supplementary Fig. S2). The Western blot revealed weaker bands at approximately 70 kDa in six out of eight IgM crispants relative to the controls.

## Discussion

In the present study, we explored the possibilities for generating an IgM^+^ B cell-deficient Atlantic salmon by CRISPR/Cas9-mediated knockout of IgM. Originally, two gRNAs targeting the µ1 region of both IgM A and B genes were designed and tested in a pilot experiment, with only one gRNA producing sufficient mutagenesis. The IgM gRNA that did produce mutations was therefore used to create the fish described in this study. Variable gRNA efficiency is well known in the field of CRISPR/Cas gene editing, and although tools have been developed to optimize gRNA design (e.g., CHOPCHOP^26^ or CRISPick^27^), predicting the in vivo efficiency remains difficult. A study in zebrafish which compared the in vivo editing efficiencies of 50 gRNAs targeting 14 genes with predictions from seven commonly used gRNA design tools, revealed significant discrepancies between predicted and actual outcomes^28^. Our sequencing data revealed an average of 97% mutagenesis of both IgM loci in the IgM crispants, with nearly 80% frameshift mutation variants. The editing efficiency observed is comparable to or higher than what we have reported previously using CRISPR/Cas9 in the F0 generation^13,24,29–32^.

Our results on mRNA and protein level suggests phenotypic effects of the CRISPR-induced mutagenesis of IgM, as we found lower mRNA expression of membrane-bound IgM in head kidney and spleen of the IgM crispants compared to the controls, as well as a large reduction in IgM^+^ B cells from peripheral blood in the crispants. The average IgM^+^ B cell frequency in the control group was 10.2%. In contrast, the average number of IgM^+^ B cells in the IgM crispants was 2.3%, representing an approximate 78% reduction. CRISPR-based gene editing usually leads to a high degree of mosaicism in the organism edited. The persistence of both membrane-bound IgM mRNA and IgM^+^ B cells, despite the observed reduction, is therefore likely explained by the retention of non-edited (i.e., wild-type) DNA or in-frame mutation variants in some of the cells that do not produce a phenotypic effect. The IgM crispants show overall normal or higher mRNA levels of secreted IgM in the spleen and head kidney, respectively. However, two individuals had highly reduced mRNA levels of secreted IgM. These individuals were also among the crispants with the highest rate of frameshift mutations. In serum, the majority of the crispants analysed had seemingly lower IgM protein levels compared to controls. It is conceivable that the remaining IgM^+^ B cells in our fish, although fewer, may still be able to proliferate, differentiate, and secrete IgM. The duplicated Ig heavy chain loci in salmon complicates achieving completely IgM^+^ B cell-depleted fish using CRISPR, as both A and B genes need to be sufficiently mutated.

To study gene function, individuals should be complete knockouts since wild-type DNA could potentially rescue the phenotype produced by incomplete mutagenesis^13^. Breeding the IgM crispants to the F1 generation is therefore desired for future studies. A remaining question is whether homozygous IgM knockout fish will survive. Considering that IgM is the predominant antibody in the systemic immune compartment^16^, it was somewhat surprising to us that the IgM crispants survived despite being reared in a non-sterile environment. In a study characterizing IgM knockout rats, homozygous knockout rats with depleted B cell levels were reported to survive, but these rats were kept under pathogen-free conditions^22^. In a study with antibody- and B cell-deficient pigs, the pigs died due to bacterial infection when reared conventionally^33^. However, these pigs were mutated in the Ig J-region gene segment, resulting in the absence of all Ig classes. It is possible that the remaining IgM^+^ B cells in our IgM crispants are sufficient for the fish to survive, and that homozygous IgM knockout fish will not. However, zebrafish Rag1 knockout fish survive in a non-sterile environment despite lacking B cells completely^34^. We are also tempted to speculate whether IgT^+^ B cells and IgT antibodies could compensate for the reduction of IgM, considering that our results indicate increased IgT mRNA levels in the spleen and head kidney of the crispants. Notably, two of the most highly mutated fish (IgM_6 and IgM_7) showed the lowest levels of IgM expression in both spleen and head kidney and were also among the highest IgT-expressing fish. However, potential compensatory effects require further research to be clarified, most particularly using IgT-specific antibodies.

CRISPR/Cas9 has previously been applied in Atlantic salmon to study or verify gene function of traits related to reproduction and fatty acid synthesis^13–15,29–31,35^. Although the technology has been used to knock out and study immune-related genes in salmonid cell lines^36–38^, this study marks the first report of an immune-related gene knockout in this species in vivo. Given the common occurrence of mosaicism in CRISPR-edited organisms, breeding these F0 IgM crispants to establish homozygous frameshift-mutated fish is desired to generate a completely IgM^+^ B cell knockout fish that can be used in future studies. The generation of IgM knockout fish has valuable scientific purposes, particularly in advancing our understanding of the immune system in Atlantic salmon. The B cells and antibodies play a central role in the adaptive immune responses, while natural IgM contributes to the innate humoral immune response through functions such as opsonization, neutralization, and complement activation. A (challenge) model with fish lacking these immune mechanisms offers a unique opportunity to further elucidate the role of IgM as well as the contributions of non-IgM components to immunity. Furthermore, since the humoral adaptive responses predominately orchestrated by B cells and antibodies are considered crucial for prophylactic immunity, the same model may possibly offer insights with significant value for vaccine development.

## Methods

### gRNA and Cas9 mRNA synthesis

Gene sequences for IgM (locus A (LOC106606767) and locus B (GU129140)) were obtained from the Atlantic salmon reference genome assembly (ICSASG_v2) on the National Center for Biotechnology Information (NCBI) website. Target sites with the protospacer adjacent motif (PAM) sequence 5′-NGG-3′ for Cas9 recognition were identified using Geneious Prime software (v. 11.0.12). Candidate gRNAs were chosen from the first exons, and a BLAST^39^ search was conducted to screen for gRNAs with minimal off-target effects. Two gRNAs were selected, which targeted the µ1 exon of the Ig heavy chain Mu region in both locus A and B (IgM-1: 5’-GCGCAATGTGGCTCCGGGAC-3′, IgM-2: 5′-CTGGCTTCACGCCTGCCTCC-3′). The gRNAs were synthesized based on^40^ as described previously^41^. Oligos for gRNA synthesis were ordered from Eurofins Genomics. Cas9 mRNA was synthesized as described previously^24^.

### Generation of IgM crispants

Salmon eggs and sperm were provided by Mowi (Askøy, Hauglandshella, Norway). Fertilization and CRISPR/Cas9 injections were performed as described in^41^, using 50 ng/µL gRNA and 150 ng/µL Cas9 mRNA. The gRNA efficiencies were tested in a pilot experiment where only one showed efficient mutagenesis (data not shown). This gRNA was therefore used to generate the IgM crispants mentioned in the current paper. gRNA targeting the pigmentation gene *slc45a2* was included in the injection mix since disruption of this gene results in an albino or albino-mosaic pigmentation phenotype^24^. This phenotype serves as a marker and allows visual identification of individuals where the injection mix has been delivered successfully^13,24^. Two control groups were also included: one group of eggs injected with gRNA targeting *slc45a2* only (albino control) and one group of non-injected eggs (wild-type control). The eggs were incubated at 6 °C in a hatchery until reaching the feeding stage. After reaching the feeding stage, the fish were maintained in freshwater under a constant light regime and fed commercial dry feed (Skretting).

### Sampling

When the fish reached approximately 100–200 g, individuals from the IgM crispant group displaying an albino or mosaic phenotype were selected for sampling. These fish, along with a group of albino and wild-type control fish, were sampled to assess the mutation frequency and potential phenotypic effects of the CRISPR-mediated IgM knockout. In total 40 fish were sampled, distributed as 20 IgM crispants and 20 controls (10 albino and 10 wild-type). The fish were euthanized with a lethal dose of tricaine (MS-222). Weight and length measurements were recorded during the sampling process. Tissue from the adipose fin was collected and preserved in ethanol for DNA sequencing. Blood was collected from the caudal vein of the fish and transferred into sterile 1.5 mL tubes for subsequent Western blot analysis of serum samples. In addition, from six IgM crispants (with at the time unknown mutation efficiency) and six controls (three albino and three wild-type), blood was also collected for flow cytometry analysis, as well as DNA sequencing of isolated leukocytes. Here, approximately 1 mL of the blood was transferred into tubes containing 4 mL transport medium, consisting of Leibovitz (L-15) medium (Gibco, Life Technologies) supplemented with penicillin (100 units/mL) and streptomycin (100 µg/mL) (P/S), fetal bovine serum (2%), and heparin (20 IE/mL) as described previously^42^. Finally, head kidney and spleen tissues were collected and preserved in RNAlater™ stabilization solution (ThermoFisher Scientific) for gene expression analyses.

### Leukocyte isolation

Peripheral blood leukocyte (PBL) isolation was performed as described previously^42,43^ Whole blood suspension in transport medium was carefully loaded onto a 54% Percoll solution (GE Healthcare) and centrifuged at 400 × g for 40 min at 4 °C. The interface was collected and washed twice in L-15 medium with P/S, with centrifugation in between. A portion of isolated PBLs were stained with Trypan Blue (Gibco) and counted using a Countess Automated Cell Counter (Invitrogen). The leukocyte suspension was centrifuged and the resulting pellet was resuspended in PBS containing 0.1% fetal calf serum (PBS^+^) to a final concentration of 20,000 cells/µL. These cells were stained for surface IgM analysis via flow cytometry, while remaining cells (~ 1 million cells) were stored frozen for DNA sequencing. Unless otherwise indicated, all centrifugation steps were performed at 500 × g for 10 min and at 4 °C.

### DNA sequencing of fin tissue and leukocytes

Genomic DNA was extracted from fin tissue and PBL (1 × 10^6^ cells) using the DNeasy Blood & Tissue Kit (Qiagen) following the manufacturer’s instructions. Sequencing libraries were prepared using a two-step PCR protocol adapted from^40^ as described previously^44^. Briefly, in the first PCR gene-specific primers are used to amplify a region covering the gRNA target site. These primers contain adapters facilitating sample barcoding during the second PCR. The primer sequences used for amplification of the target sites are listed in Supplementary Table S6. The libraries were sequenced on the Illumina MiSeq platform using the MiSeq Kit v3 with 300 bp paired-end reads. Fastq files were filtered and trimmed using Cutadapt (v. 2.8)^45^: low quality bases were trimmed from the 3’ end of the reads (-q 20) and reads shorter than 100 bp were discarded (-m 100). Sequence reads retained after filtering were mapped to the reference sequences of the respective target genes using Muscle (v. 3.8.1551)^46^. Custom python scripts were used to analyze the reads. The reads were categorized into three groups: WT (representing wild-type reads having a perfect match to the reference sequence), in-frame (reads with insertions or deletions that result in an in-frame mutation, e.g., a 3, 6 or 9 nt insertion or deletion), and frameshift (reads with insertions or deletions that result in a frameshift mutation). The number of reads in the respective group was divided by the number of total reads to calculate the ratio of WT, in-frame, and frameshift reads in each individual.

### RT-qPCR of head kidney and spleen tissues

Total RNA was isolated from head kidney and spleen using the RNeasy Mini Kit (Qiagen) according to the manufacturer’s instructions. cDNA was synthesized from RNA (500 ng input) using the SuperScript™ VILO™ cDNA Synthesis Kit (ThermoFisher Scientific) in 10 µL reactions. RT-qPCR assays for the immune-related genes IgM (secreted and membrane-bound, sIgM and mIgM), IgT, and reference gene Elongation factor-1 alpha (Ef1a), were set up in duplicate 7 µL reactions containing 10 ng cDNA equivalents of RNA, 400 nM forward and reverse primers, 29 nM ROX reference dye, and 1X Brilliant III Ultra-Fast SYBR Green qRT-PCR Master Mix. The reactions were run on the QuantStudio™ 5 Real-Time PCR System (Applied Biosystems) using the following conditions: 95 °C for 3 min, 40 cycles of 95 °C for 5 s and 60 °C for 20 s, followed by melt curve analysis. The Pfaffl method was used to calculate the relative gene expression (fold change) of the genes of interest (GOI). Individual samples were normalized by subtracting the Ct value of the reference gene from the GOI. The ΔCt average of the control samples (albino and wild-type) was used as the calibrator for each GOI. The ΔΔCt was calculated by subtracting the calibrator from the ΔCt, and finally, the relative gene expression was calculated. Primers used and their efficiencies for the different tissues are listed in Table 2. The gene expression data was statistically analysed using the Mann–Whitney test, an unpaired, non-parametric test, in GraphPad Prism (v. 10.3.1).

**Table 2:** Primer sequences and efficiencies used in RT-qPCR immune gene expression analyses of head kidney (HK) and spleen tissue dissected from gene-edited Atlantic salmon (CRISPR-mediated knockout of Immunoglobulin M (IgM) and control fish.

Target gene	Forward primer (5′-3′)	Reverse primer (5′-3′)	Accession	Efficiency (HK/Spleen)	References
Ef1a	CACCACCGGCCATCTGATCTACAA	TCAGCAGCCTCCTTCTCGAACTTC	AF321836.1	2.05/2.09	Ytteborg et al.^47^
sIgM	CTACAAGAGGGAGACCGGAG	AGGGTCACCGTATTATCACTAGTT	XM_014203125.1	2.05/2.02	Tartor et al.^48^
mIgM	CCTACAAGAGGGAGACCGA	GATGAAGGTGAAGGCTGTTTT	Y12457	2.06/2.05	Tartor et al.^48^
IgT	CAACAAAGTCACTGTCACCTGGAA	CCGTCAGCGGTTCTGTTTTG	GQ907003.1	2.04/2.02	Nunez-Ortiz et al.^49^

### Flow cytometry analysis of surface IgM^+^ B cells in PBL

PBLs from individual fish (1 × 10^6^ cells/well) were transferred into a 96-well round-bottom plate for staining and subsequent flow cytometry analysis as previously described^43^. Monoclonal anti-Salmon IgM antibody (F1-18), which was kindly provided by Dr. Karsten Skjødt^25^, recognizes the µ3 region of both IgM A and B isotypes. This primary antibody was added to the cells at a 1:200 dilution and incubated for 30 min. F(ab’)2-Goat anti-Mouse IgG, IgM (H + L)-Alexa Fluor™ 488 (Invitrogen) was used as a secondary antibody (1:500 dilution) and incubated together with Fixable Viability Dye 780 (FVD780, eBioscience) at a 1:1000 dilution for 30 min. The cells were washed twice with PBS^+^ in between staining. Finally, stained cells were washed once with PBS^+^ and resuspended in 150 µL PBS^+^. All steps were carried out in the dark and on ice, and centrifugation was done at 500 × g for 10 min at 4 °C.

Flow cytometry analysis of surface IgM^+^ peripheral blood B cells was performed on a FACSCanto II flow cytometer (Becton Dickinson). Data were analysed with FlowJo software (v. 10.8.1) using the following gating strategy: doublets were excluded and the remaining single cell population was gated for viability (FVD780^-^). Within the viable leukocyte population, cells were further gated based on their forward scatter (FSC) and side scatter (SSC) profiles. The FCS^low^ SCC^low^ subpopulation was identified as the lymphocyte gate. The proportion of viable lymphocytes expressing surface IgM was then determined based on Alexa 488 fluorescence emission. Further details can be found in Supplementry Fig. S3.

### Total IgM in serum by Western blot

Blood samples were left to clot at 4 °C overnight and then centrifuged for 5 min at full speed in a tabletop centrifuge. Western blot analysis of IgM protein in serum was performed based on Jenberie et al.^50^ but with some modifications: total protein content in serum was determined using a Pierce BCA Protein assay (ThermoFisher Scientific). Total protein (500 ng input) was mixed with 1X DTT Reducing Agent (Cell Signaling) and 1X Blue Loading Buffer (Cell Signaling) and denatured at 95 °C for 5 min before loading onto 4–20% Mini-PROTEAN® TGX™ Precast Protein Gels (Bio-Rad Laboratories). The samples were subjected to SDS-PAGE with 1X Tris–Glycine-SDS running buffer for 1 h 30 min at 100V and blotted onto 0.45 µM nitrocellulose membranes (Bio-Rad Laboratories). The membranes were blocked with 5% dry milk in 1X TBST buffer for 1 h at room temperature and incubated with F1-18 antibody^25^ (1:200) overnight. After three washes, the samples were incubated with HRP-conjugated anti-Mouse IgG (1:2000) for 1 h 30 min and developed using SuperSignal™ West Femto Maximum Sensitivity Substrate (ThermoFisher Scientific). Finally, the samples were visualized using the iBright CL1000 Imaging System (Invitrogen).

## Supplementary Information

Supplementary Information 1.

Supplementary Information 2.

## Data Availability

The data supporting the findings of this study are available within the article and the supplementary information. The raw MiSeq sequencing data generated in this study is available at https://doi.org/10.6084/m9.figshare.27993167.
