# Supplementary material for: Generation of IgM+ B cell-deficient Atlantic salmon (Salmo salar) by CRISPR/Cas9-mediated IgM knockout
Source: Sci Rep. 2025 Jan 28;15:3599. doi: 10.1038/s41598-025-87658-5 (PMC11775215; doi:10.1038/s41598-025-87658-5)
Supplement: Supplementary file 1 — Supplementary Information 1. [file 41598_2025_87658_MOESM1_ESM.pdf]

Supplementary File 1: Figures

Generation of IgM^{+} B cell-deficient Atlantic salmon (*Salmo salar*) by CRISPR/Cas9-mediated IgM knockout

Mari Raudstein[1], Ma. Michelle D. Peñaranda[1], Erik Kjærner-Semb[1], Søren Grove[1], H. Craig Morton[1], Rolf Brudvik Edvardsen[1]

[1]Institute of Marine Research, Bergen, Norway

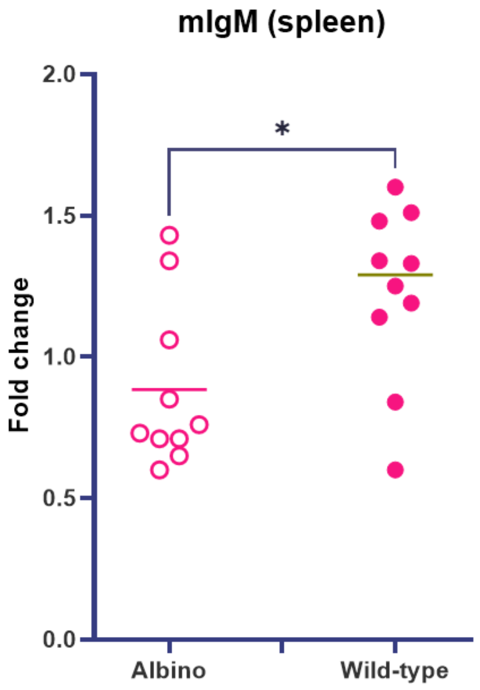

Supplementary Fig. S1 Expression of mIgM in spleen tissue from albino and wild-type control groups. The Mann-Whitney test was used for statistical analysis, $*p < 0.05$.

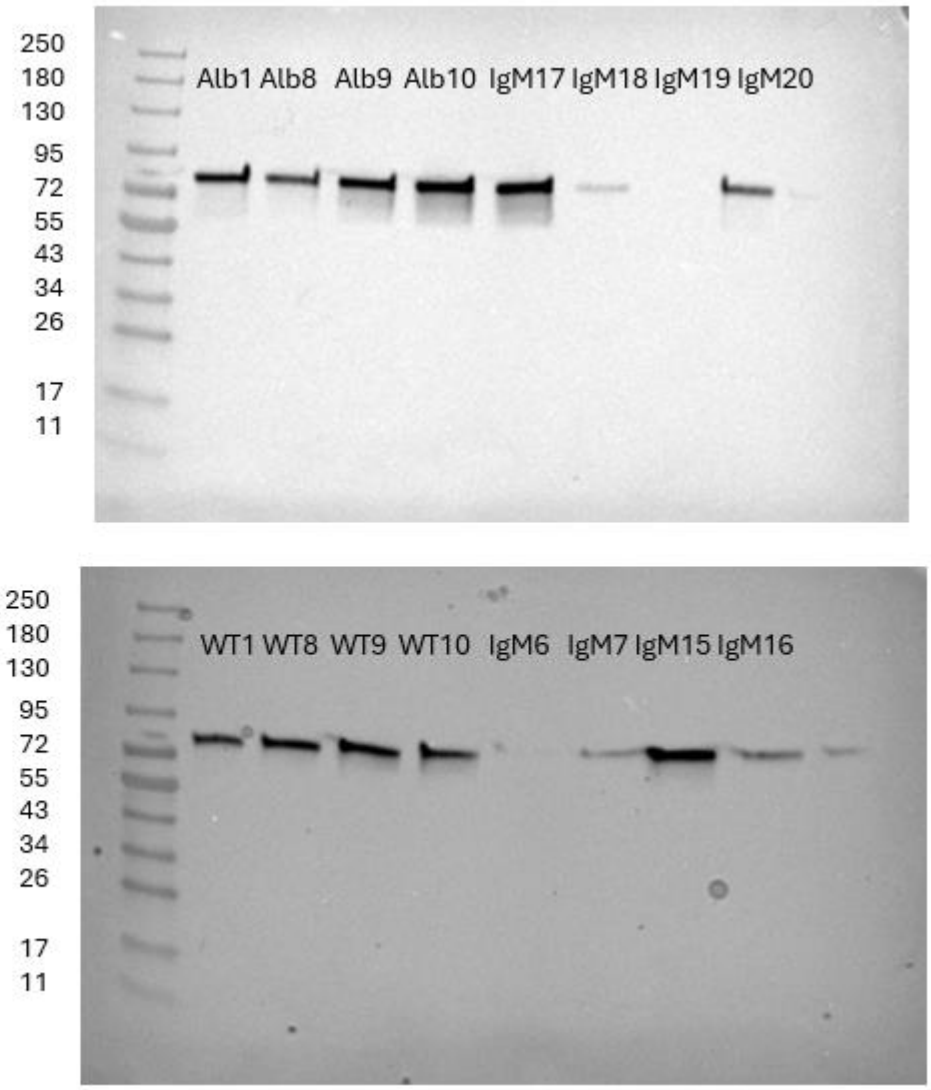

Supplementary Fig. S2 Detection of IgM protein in sera by Western blot analysis. Membranes stained with monoclonal IgM-specific antibody (F1-18) with marker overlay (kDa) are shown.

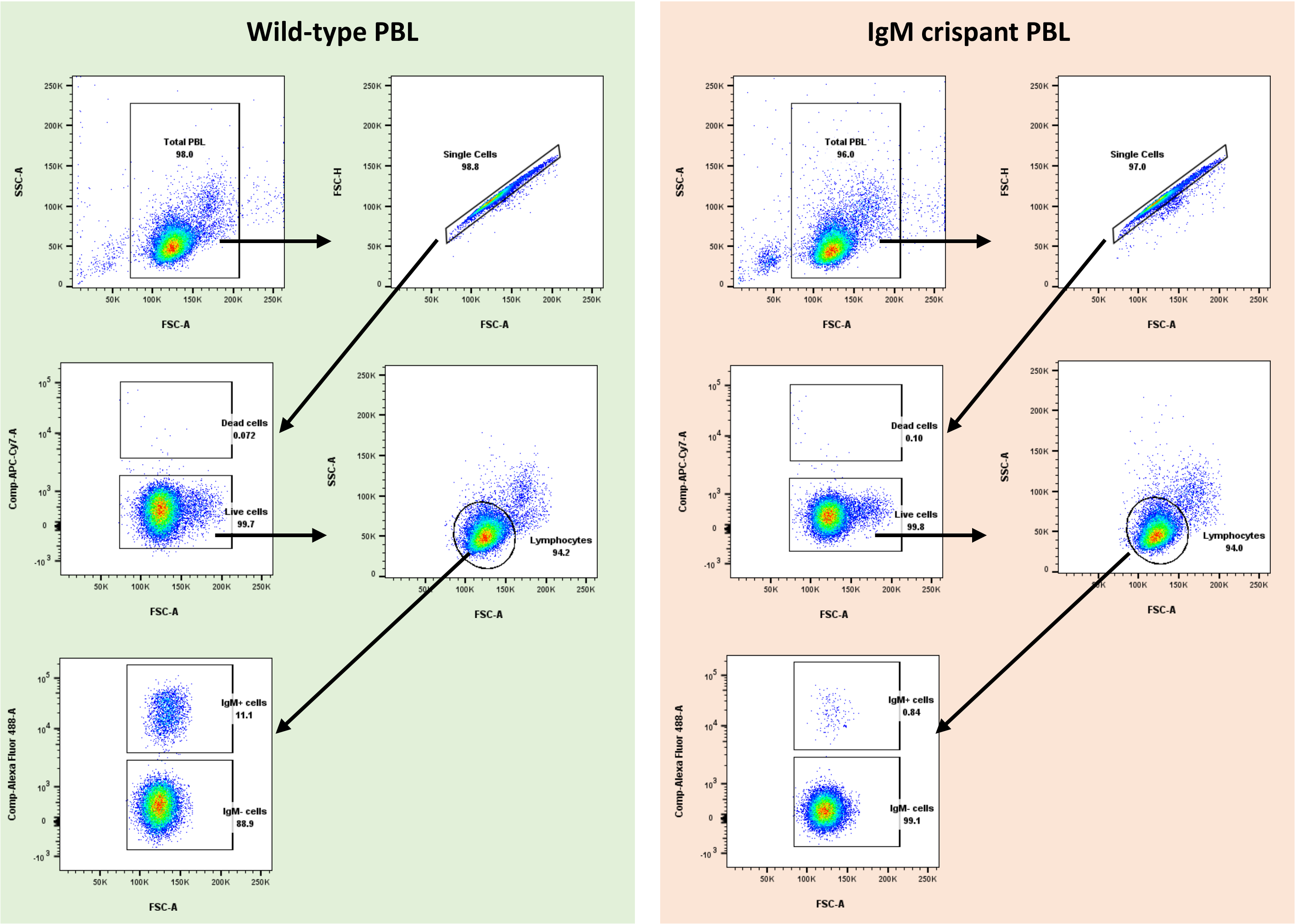

Supplementary Fig. S3 Gating of PBL for Surface IgM Expression Analysis. PBLs were collected, stained, and analysed by flow cytometry to determine the proportion of IgM^+ and IgM^- lymphocytes. Doublets were excluded from the total leukocyte population by correlating FSC-A vs. FSC-H to detect disproportions between cell sizes vs. cell signal. The remaining single-cell population was further discriminated into live ($FVD780^-$) and dead ($FVD780^+$) cells based on their FVD780 fluorescence profiles. Within the viable cell subpopulation (lower half), lymphocytes were gated using forward scatter (FSC) and side scatter (SSC) characteristics, identifying the FSC^{low} SSC^{low} subpopulation as the lymphocyte subset. The percentage of lymphocytes expressing surface IgM+ was then determined based on Alexa-488 fluorescence emission (20,000 events). Left and right panels show representative gating for wild-type vs. IgM-crispants PBL samples, respectively.
