# Supplementary material for: Generation of IgM+ B cell-deficient Atlantic salmon (Salmo salar) by CRISPR/Cas9-mediated IgM knockout
Source: Sci Rep. 2025 Jan 28;15:3599. doi: 10.1038/s41598-025-87658-5 (PMC11775215; doi:10.1038/s41598-025-87658-5)
Supplement: Supplementary file 2 — Supplementary Information 2. [file 41598_2025_87658_MOESM2_ESM.pdf]

Supplementary File 2: Tables

Generation of IgM$^+$ B cell-deficient Atlantic salmon (*Salmo salar*) by CRISPR/Cas9-mediated IgM knockout

Mari Raudstein[1], Ma. Michelle D. Peñaranda[1], Erik Kjærner-Semb[1], Søren Grove[1], H. Craig Morton[1], Rolf Brudvik Edvardsen[1]

[1]Institute of Marine Research, Bergen, Norway

Supplementary Table S1 Weight and length measurements recorded in wild-type (WT) control fish, albino (Alb) control fish, and IgM crispants.

Fish ID	Length (cm)	Weight (g)
WT_1	23	145
WT_2	19	83
WT_3	22.5	140
WT_4	20.5	101
WT_5	27.5	213
WT_6	23.9	147
WT_7	23.3	148
WT_8	22.6	106
WT_9	26.5	203
WT_10	24.4	157
Alb_1	20	112
Alb_2	25	174
Alb_3	26	204
Alb_4	22	127
Alb_5	28.5	236
Alb_6	25.8	167
Alb_7	25.8	164
Alb_8	28.3	241
Alb_9	25	152
Alb_10	30.7	306
IgM_1	24	179
IgM_2	20.5	107
IgM_3	23.5	169
IgM_4	22	129
IgM_5	21.5	128
IgM_6	23	146
IgM_7	21	117
IgM_8	21.5	129
IgM_9	23	131
IgM_10	26.2	188
IgM_11	27.8	237
IgM_12	29	271
IgM_13	30.6	305
IgM_14	30	276
IgM_15	27.4	218
IgM_16	27.6	226
IgM_17	27.5	232
IgM_18	28.1	241
IgM_19	26.7	201
IgM_20	27.4	226

Supplementary Table S2 CRISPR/Cas9-induced mutagenesis of both IgM loci (A and B) in Atlantic salmon. Mutagenesis was assessed by high-throughput sequencing of fin tissue. The percentage of sequence reads obtained from individual fish showing a perfect match to the reference sequence (no indel), in-frame or frameshift mutations.

Fish ID	IgM-A			IgM-B		
	No indel (%)	In-frame (%)	Frameshift (%)	No indel (%)	In-frame (%)	Frameshift (%)
IgM_1	0.9	1.4	97.6	7.9	27.6	64.5
IgM_2	0.7	12.3	87.1	3.7	13.7	82.6
IgM_3	1.4	19.8	78.9	2.6	13.4	84.0
IgM_4	0.4	9.1	90.6	2.0	29.7	68.4
IgM_5	6.2	16.5	77.4	4.0	15.2	80.8
IgM_6	0.2	4.2	95.6	0.4	13.7	85.9
IgM_7	1.3	28.5	70.3	1.0	1.4	97.6
IgM_8	3.2	55.9	40.9	20.5	13.8	65.7
IgM_9	4.8	18.0	77.3	1.8	19.7	78.5
IgM_10	0.8	24.8	74.4	0.5	38.5	61.0
IgM_11	8.0	20.8	71.2	0.9	18.2	80.9
IgM_12	0.4	0.3	99.3	6.3	18.0	75.7
IgM_13	0.6	34.1	65.3	0.4	36.4	63.2
IgM_14	6.3	29.3	64.4	1.2	5.0	93.9
IgM_15	1.0	17.4	81.6	0.2	35.9	63.9
IgM_16	3.8	24.1	72.1	4.0	6.9	89.1
IgM_17	0.8	21.6	77.6	1.2	17.6	81.3
IgM_18	0.3	16.2	83.5	0.8	11.5	87.6
IgM_19	0.6	9.0	90.3	0.3	39.6	60.1
IgM_20	0.3	23.0	76.8	1.6	27.3	71.1
Wild-type	99.4	0.0	0.6	99.4	0.0	0.6
Albino	99.4	0.0	0.6	99.3	0.0	0.6

Supplementary Table S3 Comparison of mutagenesis of both IgM loci (A and B) in fin and leukocyte tissues in Atlantic salmon. Mutagenesis was assessed by high-throughput sequencing. The percentage of sequence reads obtained from individual fish showing a perfect match to the reference sequence (no indel), in-frame or frameshift mutations.

Fish ID	IgM-A			IgM-B		
	No indel (%)	In-frame (%)	Frameshift (%)	No indel (%)	In-frame (%)	Frameshift (%)
IgM_15(fin)	1.0	17.4	81.6	0.2	35.9	63.9
IgM_15(WBC)	1.6	13.3	85.1	0.4	14.0	85.7
IgM_16(fin)	3.8	24.1	72.1	4.0	6.9	89.1
IgM_16(WBC)	3.7	31.5	64.8	24.4	14.6	61.0
IgM_17(fin)	0.8	21.6	77.6	1.2	17.6	81.3
IgM_17(WBC)	6.2	20.0	73.8	0.5	10.0	89.5
IgM_18(fin)	0.3	16.2	83.5	0.8	11.5	87.6
IgM_18(WBC)	0.3	17.7	82.0	5.3	17.8	76.9
IgM_19(fin)	0.6	9.0	90.3	0.3	39.6	60.1
IgM_19(WBC)	0.2	4.2	95.7	0.3	5.8	93.9
IgM_20(fin)	0.3	23.0	76.8	1.6	27.3	71.1
IgM_20(WBC)	0.2	17.7	82.1	0.3	18.5	81.2

Supplementary Table S4 Cq values used in mRNA gene expression analysis of Ig genes in IgM crispants (IgM_) and controls (Alb_ and WT_). UD is undetermined.

Fish ID	Head kidney				Spleen			
	Cq sIgM	Cq mIgM	Cq IgT	Cq Elf1a	Cq sIgM	Cq mIgM	Cq IgT	Cq Elf1a
Alb_1	19.96	22.93	24.7	19.49	17.67	23.66	23.97	20.38
Alb_2	20.27	23.3	23.8	19.98	19.63	24.53	24.57	20.84
Alb_3	21.82	25.15	27.35	21.86	17.87	24.27	23.84	19.78
Alb_4	19.6	23.65	24.3	20.03	17.32	24.73	23.13	20.51
Alb_5	20.02	23.34	24.49	19.68	20.74	25.46	25.05	21.46
Alb_6	19.71	23.99	25.06	20.49	17.92	23.44	23.53	20.07
Alb_7	21.42	24.82	25.73	20.68	19.43	25.04	24.97	20.79
Alb_8	20.45	23.43	24.89	20.28	18.96	24.75	24.85	20.59
Alb_9	20.63	23.85	24.72	20.52	18.19	24.47	23.98	20.1
Alb_10	20.94	23.68	24.78	21	20.16	25.93	25.95	21.68
WT_1	19.19	23.22	23.9	19.14	18.41	23.34	23.96	20.11
WT_2	20.16	23.65	25.45	20.01	18.97	23.81	24.81	20.45
WT_3	21	25.18	25.44	21.07	17.99	23.8	23.87	20.61
WT_4	20.11	23.22	25.15	19.92	18.33	23.61	24.34	20.15
WT_5	20.16	24.24	25.36	20.79	17.9	23.61	23.18	20.49
WT_6	20.73	24.56	25.04	20.93	19.47	24.26	24.62	20.88
WT_7	21.21	24.14	25.4	20.43	19.75	24.29	24.12	20.7
WT_8	21.28	23.69	25.23	20.84	21.5	25.85	26.23	21.36
WT_9	21.52	25.02	26.05	21.84	18.28	24	23.86	19.98
WT_10	21.4	24.27	25.24	21.41	19.86	24.87	24.61	21.34
IgM_1	19.29	24.72	22.87	20.33	19.25	25.84	23.56	21.19
IgM_2	21.37	26.32	25.27	21.95	18.2	24.27	23.11	20.5
IgM_3	20.64	26.07	24.25	21.59	19.73	26.12	24.49	21.52
IgM_4	20.25	25.44	23.64	20.75	18.73	25.62	24.02	21.12
IgM_5	19.06	24.19	23.35	19.89	19.21	24.73	24.03	20.63
IgM_6	21.71	25.21	22.09	19.93	21.72	26.92	22.13	21.17
IgM_7	22.07	25.55	22.76	19.86	22.76	27.83	24.18	21.57
IgM_8	19.37	24.88	23.28	19.86	18.17	25.17	23.5	20.5
IgM_9	18.88	25.06	23.81	19.86	17.5	25.18	23.13	20.12
IgM_10	18.8	24.09	23.6	18.96	19.83	26.21	23.82	21.13
IgM_11	19.37	24.9	23.92	20.17	17.89	24.64	23.55	20.14
IgM_12	19.33	24.7	23.82	19.76				
IgM_13	19.36	24.14	23.28	19.85	20.74	26.3	25.22	21.97
IgM_14	19.17	24.57	23.09	19.86	18.88	24.73	22.75	20.14
IgM_15	19.36	23.38	23.57	19.77	17.13	26.27	23.94	20.5
IgM_16	19.57	23.42	23.79	19.94	19.24	25.92	24.02	20.85
IgM_17	19.39	24.92	24.16	20.06	18.86	26.32	23.84	20.66
IgM_18	18.82	24.47	23.39	19.81	18.3	26.25	23.75	20.3

IgM_19	19.21	25.07	23.11	19.79	18.47	26.09	22.81	20.08
IgM_20	19.31	24.62	23.69	20.01	17.95	25.14	22.99	20.27
NRT	UD	33.70	34.48	28.55	39.00	30.93	31.72	24.31
NC	UD	UD	UD	UD	UD	UD	UD	UD

Supplementary Table S5 IgM$^+$ B cell frequency measured by flow cytometry in IgM crispants and controls (Albino (alb) and wild-type (WT) controls). Monoclonal IgM-specific antibody (F1-18) was used to target the IgM$^+$ B cells.

Fish ID	IgM$^+$ B cells (% of leukocytes)
WT_8	4.44
WT_9	11.1
WT_10	16.3
Alb_8	8.82
Alb_9	8.37
Alb_10	12.3
IgM_15	0.84
IgM_16	4.81
IgM_17	3.19
IgM_18	1.70
IgM_19	2.22
IgM_20	1.26

Supplementary Table S6 Primers used for amplification of the Immunoglobulin heavy chain (IgHC) M (Mu) locus A and B target sites. Lowercase letters indicate MiSeq adapters.

Target	Forward primer (5'-3')	Reverse primer (5'-3')	GeneID
IgHC Mu locus A	tctttccctacacgacgctcttccgatctCTGTTAACTATTTTATTTGGTCAGTGTTGG	TggagttcagacgtgtgctcttccgatctTTACACGGAGTTGACTGACTCCC	LOC106606767
IgHC Mu locus B	tctttccctacacgacgctcttccgatctGAACTATTTTATTTGGTCAGTGTTGATTT	TggagttcagacgtgtgctcttccgatctAGCTTCCACTGGTTTGGACC	GU129140
